# Log-ratio type estimation for the finite population mean under simple random sampling without replacement with theory, simulation and application

**DOI:** 10.1038/s41598-025-29127-7

**Published:** 2025-12-06

**Authors:** Fazal Shakoor, Muhammad Atif, Hameed Ali, Abdulrahman Obaid Alshammari, Bilal Himmat, Khaled Kefi

**Affiliations:** 1 Department of Statistics, University Peshawar, Peshawar, KP Pakistan; 2 Higher Education, Archives and Libraries Department, Government of Khyber Pakhtunkhwa, Peshawar, Pakistan; 3 Department of Mathematics, College of Science, Jouf University, 72388 Sakaka, Saudi Arabia; 4 Department of Software Engineering, Faculty of Computer Science, Sayed Jamaluddin Afghani University(SJAU), Asadabad, Kunar Afghanistan; 5 Center for Scientific Research and Entrepreneurship, Northern Border University, 73213 Arar, Saudi Arabia

**Keywords:** Estimation, Auxiliary information, Bias, Efficiency, Logarithmic Estimator, PRE, Ratio Estimator, Simple Random Sampling, Engineering, Mathematics and computing

## Abstract

We propose two novel logarithmic ratio–type estimators for the finite-population mean under simple random sampling without replacement (SRSWOR). The estimators integrate a logarithmic transformation of the auxiliary variable to stabilize variance, reduce the influence of outliers, and better capture nonlinear relationships between study and auxiliary variables. We derive closed-form expressions for first-order bias and mean squared error (MSE) and obtain analytic expressions for the optimal tuning constants by direct minimization of the approximate MSE. A comprehensive numerical study, comprising five real engineering datasets and extensive Monte-Carlo simulations from multivariate normal, log-normal and gamma populations, evaluates finite-sample behavior across a range of sample sizes and correlation structures. The proposed estimators consistently reduce MSE and deliver large percent-relative-efficiency (PRE) gains relative to the classical sample mean and common competitors (empirical PREs ≈ 283; simulation PREs up to ≈ 670), with especially large and stable improvements under skewed or heavy-tailed populations. Theoretical formulas and simulation evidence align closely, showing robustness to nonlinearity and skewness while retaining simple implementation for practitioners. Results are derived under SRSWOR using first-order approximations; extensions to higher-order corrections, stratified and two-phase designs, and uncertainty in auxiliary means are recommended for future work.

## Introduction

Accurate estimation of population means from sampled data lies at the heart of survey statistics and many applied fields, from official statistics and environmental monitoring to engineering quality control and experimental sciences^1^. When using simple random sampling (SRS) to collect data on system performance or material properties, utilizing supplementary information, such as known historical measurements or operating conditions, can enhance estimator efficacy. Estimation methods such as the ratio estimator and logarithmic-ratio type estimator utilize this auxiliary data to adjust the primary estimates, reducing the mean squared error (MSE) compared to baseline classical sample means^2^. For instance, in structural health monitoring, using temperature as an auxiliary variable can improve stress estimation in materials under load^3^. For a variety of reasons, statisticians urge the incorporation of supplemental information during the estimation step. For instance, the pioneer work in this regard is due to Cochran^4^. Since auxiliary information are available to the researcher, they can be used effectively at either, the design stage or estimation stage^5,6^. Since the supplementary information explain variation in the main study variable due to their correlation, it utilizes extra information and enhances the efficiency of estimates^7^. The method of estimation of parameter rely on the nature of relation between the survey and supplementary variable. When the auxiliary variable has a positive correlation with the primary study variable the ratio-method of estimate performs efficiently^8^. Product form estimators, on the other hand, typically perform better when the regression streak crosses the origin and the connection between the study and auxiliary variables is linear and strongly negative. These considerations underscore the importance of selecting an appropriate estimator based on the nature of the statistical association, including both the direction and the structure of relationship^9–14^.

The concept of using auxiliary information to enhance estimation accuracy was first introduced by Cochran^4^, who showed that incorporating related information can substantially improve the precision of survey estimates.. Later, Bahl and Tuteja^15^, expanded on this concept by proposing exponential ratio and product-type estimators, marking a major step forward in the effective use of auxiliary variables. These estimators exploit the functional relationship between the study and auxiliary variables to obtain more efficient and accurate estimates of population parameters, especially when the variables are highly correlated. Since then, numerous researchers have expanded and refined these concepts in the field of survey sampling. Notable contributions include those by Izunobi and Onyeka^16^, Kadilar and Cingi^14^, Singh et al.^17^, Khoshnevisan et al.^18^, Onyeka et al.^19^, Singh et al.^20^, Bhushan et al., Gupta and Shabbir^21^, Azeem et al.^22^, Sher et al.^23^, Ahmad et al.^20^, and Subramani^25^ long et al.^15^. Building on this extensive body of work, the present study extends the use of auxiliary information by developing logarithmic ratio and product-form estimators, aimed at further improving the estimation of population means.

Although several ratio and product-type estimators have been developed to improve the efficiency of population mean estimation using auxiliary information, there is still a gap in understanding how to effectively combine auxiliary variables with the study variable to achieve the greatest efficiency gains. For example, most of these estimators rely on linear or conventional transformations that may not fully exploit the structure of non-linear between study and auxiliary variables. In particular, limited attention has been given to the use of logarithmic transformations within the ratio estimation framework under simple random sampling. Furthermore, existing logarithmic estimators often lack general applicability and are rarely validated on real-world engineering datasets, where non-linearity and high correlations are common. Thus, there is a need to develop a more flexible and efficient estimator that integrates logarithmic transformation with ratio-type estimation and to evaluate its performance through theoretical comparison and empirical validation^16^.

### Novelty and significance

This study proposes a logarithmic ratio-type estimator for the population mean under simple random sampling without replacement (SRSWOR), specifically designed for engineering applications where relationships between study and auxiliary variables are complex and intricate. By applying a logarithmic transformation to the auxiliary variable, the estimator linearizes variation e.g. $Y\approx \alpha {X}^{\beta }$, stabilises variance, reduces the influence of outliers which causes nonlinearity. We derive closed-form expressions for the bias and mean square error (MSE) up to the first-order approximation under SRSWOR, and conduct a numerical assessment, based on the correlation, coefficients of variation, and skewness of the auxiliary variable, to evaluate the efficiency gains of the proposed estimator obtained through the logarithmic transformation of the auxiliary variable. Finite-sample performance is assessed through Monte Carlo experiments that emulate engineering sampling conditions (varying sample size and correlation levels) and also validated empirically on real engineering datasets. The estimator’s simplicity, theoretical grounding, and robustness make it a readily applicable improvement for mean estimation in engineering quality control, monitoring, and experimental studies, with straightforward extensions to stratified and two-phase sampling designs.

The structure of the paper is as follows:

The remainder of the paper proceeds as follows. Section 2 formalizes notation, recalls relevant classical estimators, and sets up the linearization framework under SRSWOR. Section 3 introduces the two proposed logarithmic ratio–type estimators and develops their first-order bias and MSE; closed-form optimal constants are derived there. Section 4 discusses analytic efficiency conditions and compares the new forms with established estimators. Section 5 presents empirical evaluations on five engineering datasets and a comprehensive Monte Carlo study across Normal, Lognormal, and Gamma populations. Finally, Sect. 6 summarizes the implications, limitations, and practical recommendations for applying the proposed estimators in engineering and survey practice.

## Methodology

Examine a random sample of n units selected from a population of $\left(N={N}_{1}, {N}_{2},\dots {N}_{i}\right)$ units using the simple random sampling without replacement (SRSWOR) framework. Assuming that y is the research variable and x is the auxiliary variable, a sample of size n is chosen from a population of size N using simple random sampling without replacement (SRSWOR). Assuming that the population mean μₓ of the auxiliary variable is known, denote the study variable by y and the auxiliary variable by x. To enhance the estimation of the population, mean of the research variable y, the estimators consider data from the auxiliary variable. It aims to decrease sampling error and increase precision by fusing the sample statistics with the known population mean of the auxiliary variable x assuming that the auxiliary variable’s population mean, ${\mu }_{x}$, is known.

Let us define the sampling error as follows:

$${\mu }_{x}=\frac{1}{N}\sum_{i=1}^{N}\left({X}_{i}-\mu \right)$$ and $${\mu }_{y}=\frac{1}{N}\sum_{i=1}^{N}\left({Y}_{i}-\mu \right),$$

${e}_{0}=\frac{\overline{y }-{\mu }_{y}}{{\mu }_{y}}$, and ${e}_{1}=\frac{\overline{x }-{\mu }_{x}}{{\mu }_{x}}$, such that $E({e}_{0})$ = $E\left({e}_{1}\right)=0$, $E\left({e}_{0}^{2}\right)=\frac{V\left(\overline{y }\right)}{{\mu }_{y}}$ = $\left( {\frac{{1 - n}}{n}} \right)\frac{{S_{y}^{2} }}{{\mu _{y}^{2} }}$ , and $E\left({e}_{1}^{2}\right)=\frac{V\left(\overline{x }\right)}{{\mu }_{x}}$ = $\left( {\frac{{1 - n}}{n}} \right)\frac{{S_{x}^{2} }}{{\mu _{x}^{2} }}$, and $E\left( {e_{0} e_{1} } \right) = \left( {\frac{{1 - n}}{n}} \right)\frac{{S_{{yx}} }}{{\mu _{y} \mu _{x} }}.$

### Existing estimators

The sample statistics and the known population mean of the auxiliary variable are combined to create the classical estimator. thereby aiming to reduce the sampling error and enhance estimation precision, the classical is given in the following Eq. (1) as:

1 $$\frac{{\mathop \sum \nolimits_{i = 1}^{n} y_{i} }}{n} = T_{0} \,\,MSE\left( {T_{0} } \right) = {\uplambda }\overline{Y}^{2} C_{y}^{2}$$

where $\lambda$ is the $fpc$ (finite population correction factor) given by:

$$\uplambda =\frac{1-f}{n}.$$

The classic ratio-type estimator was first presented by Cochran (Cochran, 1940) and is represented as follows in Eq. (2):

$$T_{1} = \mu_{x} \frac{{\overline{y}}}{{\overline{x}}}$$

The estimator *t* is biased for the population mean μ*x*, and its bias and MSE are determined up to the first-order approximation may be written as follows, where *y* and *x* stand for the sample means of the study and auxiliary variables, respectively:

2 $$\begin{gathered} Bias\left( {T_{1} } \right) = \left( {\frac{{1 - f}}{n}} \right)\left( {\frac{1}{{\mu _{x} }}} \right)\left( {DS_{x}^{2} - S_{{xy}} } \right) \hfill \\ MSE\left( {T_{1} } \right) \approx \left( {\frac{{1 - f}}{n}} \right)\left( {S_{y}^{2} + DS_{x}^{2} - 2DS_{{xy}} } \right) \hfill \\ \end{gathered}$$

where ${S}_{y}^{2}= \frac{1}{N-1}\sum_{i=1}^{N}{\left({Y}_{i}-{\mu }_{y}\right)}^{2}$ is population variance of y, ${S}_{x}^{2}= \frac{1}{N-1}\sum_{i=1}^{N}{\left({X}_{i}-{\mu }_{x}\right)}^{2}$ is the population variance of x, ${S}_{yx}= \frac{1}{N-1}\sum_{i}^{N}\left({X}_{i}-{\mu }_{x}\right)\left({Y}_{i}-{\mu }_{y}\right)$ is the population covariances of x and y, and ${\text{n}} = \frac{n}{N}$ and $\text{D}= \frac{{\mu }_{x}}{{\mu }_{y}}$ , represent the sampling fraction and population ratios, respectively.

The product type estimator in the context of utilizing single auxiliary variables, suggested by Subramani and Kumarapandiyan^14^, and is given as follows:

$${T}_{2}=\overline{y }\frac{\overline{x}}{{\mu }_{x}}$$

With MSE given by:

3 $$MSE\left( {{\text{T}}_{2} } \right) = \left( {\frac{{1 - n}}{n}} \right)\bar{Y}^{2} (C_{y}^{2} + C_{x}^{2} + 2\rho C_{y} C_{x} )$$

Likewise, a single exponential ratio-type estimator for the population mean ${\mu }_{y}$​ was proposed by Bahl and Tuteja^17^ and is defined as follows:

$${\text{T}}_{3}=\overline{y }exp\left(\frac{{\mu }_{x}-\overline{x}}{{\mu }_{x}+\overline{x} }\right)$$

The bias and mean square error of the exponential ratio-type estimator ${\overline{\text{y}} }_{\text{p}}$, which is biased for ${\mu }_{y}$, with MSE given by:

4 $$MSE\left( {T_{3} } \right) = \left( {\frac{{1 - n}}{n}} \right)\left( {S_{y}^{2} + \frac{1}{4}D^{2} S_{x}^{2} - DS_{{xy}} } \right)$$

Similarly, Gupta and Shabbir^18^ developed the difference type regression estimator given by:

$${\text{T}}_{4}=\overline{y }+{\varphi }_{1}\left({\mu }_{x}-\overline{x }\right)$$

where ${\varphi }_{1= }\frac{{s}_{yx}}{{s}_{x}^{2}}$​​ is the coefficient of simple regression linked to ${\beta }_{1= }\frac{{S}_{yx}}{{S}_{x}^{2}}$ and the population mean. The MSE of regression estimator are presented in Eq. (5).

5 $$MSE\left( {T_{4} } \right) = \left( {\frac{{1 - n}}{n}} \right)C_{y}^{2} \left( {1 - \rho ^{2} } \right)$$

The pioneer work on the use of log-auxiliary variable in mean estimation was due to Izunobi and Onyeka^19^, which is given by:

$${\text{T}}_{5}=\frac{{\mu }_{y}Ln(\overline{y })}{Ln\left(\overline{x }\right)}$$, $$Ln\left({\mu }_{x}\right)\ne 0$$ and $$Ln\left(\overline{x }\right)\ne 0$$

With MSE given by:

6 $$MSE\left({\text{T}}_{5}\right)= \left(\frac{1-f}{n}\right)\left({S}_{y}^{2}+{k}^{2}{D}^{2}{S}_{x}^{2}-2kD{S}_{yx}\right)$$

where $$k=\frac{1}{Ln\left({\mu }_{x}\right)}$$

The innovative use of log-transformed auxiliary variable was found in work by Mishra et al^20^. by developing the following mean estimators:

$${T}_{6}=\overline{y }+\alpha log\left(\frac{\overline{x} }{\overline{X} }\right),$$

$${T}_{7}=\overline{y }\left({w}_{1}+1\right)+{w}_{2}log\left(\frac{\overline{x} }{\overline{X} }\right),$$

With MSEs respectively given by:

7 $$MSE\left({T}_{6}\right)={Y}^{2}\theta {C}_{y}^{2}\left(1-{\rho }^{2}\right).$$

8 $$MSE{\left({T}_{7}\right)}_{min}=C+\left(\frac{B{C}^{2}+A{D}^{2}-2CDE}{{E}^{2}-AB}\right).$$

where $$\alpha =-\left(\frac{\overline{Y}\rho {C }_{y}}{{C}_{x}}\right)$$, $$A={\overline{Y} }^{2}\theta {C}_{y}^{2},$$ $$B=\theta {C}_{x}^{2}$$, $$C={\overline{Y} }^{2}\theta {C}_{y}^{2}$$, $$D=\overline{Y}\theta \rho {C }_{y}{C}_{x}$$, $$E=\overline{Y }\theta \left(\rho {C}_{y}{C}_{x}-\frac{{C}_{x}^{2}}{2}\right).$$

On the same line most recently Singh and Tiwari^21^, introduce the following adapted mean estimator in SRSWOR scheme:

$${T}_{8}={K}_{1}log\left[\frac{\overline{X }+Md}{\overline{x }+Md}\right]+{K}_{2}log\left[\frac{\overline{x }+Md}{\overline{X }+Md}\right],$$

With MSE given by:

9 $$MSE\left({T}_{8}\right)=\left[A+\left(\frac{B{E}^{2}+C{D}^{2}+2DEF}{{F}^{2}-BC}\right)\right].$$

where

$${K}_{1opt}=\frac{EF-CD}{{F}^{2}-BC}and {K}_{2opt}=\frac{BE-DF}{{F}^{2}-BC}.$$

Although the effectiveness of parameter estimates is much increased by the logarithmic-ratio type estimators, log-ratio estimates still require improvement, which is the main goal of our work. The development of enhanced log-ratio type estimators of finite population mean under the SRSWOR scheme is the focus of the following section.

## Proposed class of estimators

Taking motivation from Izunobi and Onyeka^19^, Sher et al.^22^, and Eisa et al.^1^, Under SRSWOR, two estimators of the logarithmic ratio type are created for the finite population mean.

10 $${T}_{P1}=\left({k}_{1}\overline{y }+{k}_{2}\right)\left(\frac{{\mu }_{x}}{\overline{x }-{\mu }_{x}}\right)Ln\left(\frac{\overline{x}}{{\mu }_{x}}\right)$$

11 $$T_{{P2}} = \left( {k_{3} \bar{y} + k_{4} } \right)Ln\left( {\frac{{\bar{x}}}{{\mu _{x} }}} \right)\exp \left( {\frac{{\bar{x} - \mu _{x} }}{{\bar{x} + \mu _{x} }}} \right)$$

The estimator increases the estimate process’s efficiency by employing the auxiliary variable’s logarithmic transformation, particularly in cases when there is a nonlinear relationship between the study and auxiliary variables.

### Asymptotic properties of the proposed estimators

Rewriting Eq. (10) and (11) in term of error due to sampling, given in "Methodology", as following:

12 $${T}_{P1}=\left({k}_{1}\overline{Y }\left(1+{e}_{0}\right)+{k}_{2}\right)\left(\frac{{\mu }_{x}}{\overline{X }\left(1+{e}_{1}\right)-{\mu }_{x}}\right)Ln\left(\frac{\overline{X }\left(1+{e}_{1}\right)}{{\mu }_{x}}\right),$$

13 $$T_{{P2}} = \left( {k_{3} \bar{Y}\left( {1 + e_{0} } \right) + k_{2} } \right)Ln\left( {\frac{{\bar{X}\left( {1 + e_{1} } \right)}}{{\mu _{x} }}} \right){\text{ }}\exp \left( {\frac{{\bar{X}\left( {1 + e_{1} } \right) - \mu _{x} }}{{\bar{X}\left( {1 + e_{1} } \right) + \mu _{x} }}} \right)$$

Expanding the series and after simplification of Eqs. (12) and (13), we obtain the following:

$$T_{P1} \approx k_{1} \mu_{y} - \frac{1}{2}k_{1} \mu_{y} e_{1} + \frac{1}{3}k_{1} \mu_{y} e_{1}^{2} + k_{1} \mu_{y} e_{0} - \frac{1}{2}k_{1} \mu_{y} e_{0} e_{1} + k_{2} - \frac{1}{2}k_{2} e_{1} + \frac{1}{3}k_{2} e_{1}^{2} ,$$

$$T_{{P2}} \approx k_{3} \mu _{y} e_{1} + k_{3} \mu _{y} e_{0} e_{1} - \frac{1}{8}k_{3} \mu _{y} e_{1}^{2} - \frac{1}{8}k_{3} \mu _{y} e_{0} e_{1}^{2} + k_{4} e_{1} - \frac{1}{8}k_{4} e_{1}^{2} + \left( {k_{3} \mu _{y} + k_{4} } \right)e_{1}$$

we subtract the population mean from each estimator, express the resulting deviations in terms of sampling errors, perform a Taylor expansion up to first order, and then take expectations to obtain the bias.

14 $$\left[{T}_{P1}- {\mu }_{y}\right]\approx {k}_{1}{\mu }_{y}-{\mu }_{y}-\frac{{k}_{1}{\mu }_{y}{e}_{1}}{2}+\frac{{k}_{1}{\mu }_{y}{e}_{1}^{2}}{3}+{k}_{1}{\mu }_{y}{e}_{0}-\frac{{k}_{1}{\mu }_{y}{e}_{0}{e}_{1}}{2}+{k}_{2}-\frac{{k}_{2}{e}_{1}}{2}+\frac{{k}_{2}{e}_{1}^{2}}{3},$$

15 $$\left[ {T_{P2} - { }\mu_{y} } \right] \approx k_{3} \mu_{y} e_{1} + k_{3} \mu_{y} e_{0} e_{1} + k_{4} e_{1} - 1/8\left( {k_{3} \mu_{y} e_{1}^{2} + k_{3} \mu_{y} e_{0} e_{1}^{2} + k_{4} e_{1}^{2} } \right) + \left( {k_{3} \mu_{y} + k_{4} } \right)e_{1} - \mu_{y} ,$$

Taking expectation of (14) and (15), to obtain Biases of estimator T_P1_ and T_P2_ respectively, as given by:

16 $$Bias\left({T}_{P1}\right)\approx \frac{1}{3}\uplambda {C}_{x}^{2}\left({k}_{1}{\mu }_{y}-{k}_{2}\right)-\frac{{k}_{1}}{2}{\mu }_{y}\lambda {C}_{yx}$$

17 $$Bias\left( {T_{{P2}} } \right) \approx - \frac{1}{8}\lambda C_{x}^{2} \left( {k_{3} \mu _{y} - k_{4} } \right)$$

Squaring both side of Eqs. (14) and (15), and after simplification, we get:

18 $$E[T_{P} 1 - \mu _{y} ]^{2} \approx E[k_{1} \mu _{y} - \mu _{y} - (k_{1} \mu _{y} e_{1} )/2 + (k_{1} \mu _{y} e_{1}^{2} )/3 + k_{1} \mu _{y} e_{0} - (k_{1} \mu _{y} e_{0} e_{1} )/2 + k_{2} - (k_{2} e_{1} )/2 + (k_{2} e_{1}^{2} )/3]^{2}$$

19 $$E\left[ {T_{P2} - { }\mu_{y} } \right]^{2} \approx E\left[ {k_{3 } \mu_{y} e_{1} + k_{3} \mu_{y} e_{0} e_{1} + k_{4} e_{1} - \frac{1}{8}\left( {k_{3} \mu_{y} e_{1}^{2} + k_{3} \mu_{y} e_{0} e_{1}^{2} + k_{4} e_{1}^{2} } \right) + \left( {k_{3} \mu_{y} + k_{4} } \right)e_{1} - \mu_{y} } \right]^{2}$$

Which, after simplifications, we obtain:

20 $$MSE\left( {T_{P1} } \right) \approx k_{1}^{2} \mu_{y}^{2} A_{1} - 2k_{1} \mu_{y}^{2} C_{1} - 2k_{1} k_{2} \mu_{y} E_{1} + k_{2}^{2} B_{1} - 2k_{2} \mu_{y} D_{1} + \mu_{y}^{2}$$

21 $$MSE\left( {T_{P2} } \right) \approx E\left[ {\left( {T_{P2} - \mu_{y} } \right)^{2} } \right] \approx k_{3}^{2} \mu_{y}^{2} A_{2} - 2k_{3} \mu_{y}^{2} C_{2} - 2k_{3} k_{4} \mu_{y} E_{2} + k_{4}^{2} B_{2} - 2k_{4} \mu_{y} C_{2} + \mu_{y}^{2}$$

where,

$${A}_{1}=1+\lambda {C}_{y}^{2}+\frac{11}{12}\lambda {C}_{x}^{2}-2\lambda {C}_{x}{C}_{y}$$,$${B}_{1}=1+\frac{11}{12}\lambda {C}_{x}^{2}$$, $${C}_{1}=1+\frac{1}{3}\lambda {C}_{x}^{2}-\lambda {C}_{x}{C}_{y}$$, $${D}_{1}=1+\frac{1}{3}\lambda {C}_{x}^{2}$$, and $${E}_{1}=1+\frac{11}{12}\lambda {C}_{x}^{2}-\lambda {C}_{x}{C}_{y}$$, $${A}_{2}=\frac{\alpha \hspace{0.17em}\left(1+\beta \right)\hspace{0.25em} +\hspace{0.25em} {\alpha }^{2}}{64},{B}_{2}=\frac{\alpha \hspace{0.25em} +\hspace{0.25em} {\alpha }^{2}}{64},{E}_{2}=\frac{\alpha \hspace{0.25em} +\hspace{0.25em} {\alpha }^{2}}{32},{C}_{2}=\frac{\alpha }{4},\boldsymbol{}\alpha =\uplambda {C}_{x}^{2},and\upbeta =\lambda {C}_{y}^{2}$$.

To minimize MSE of T_{P1}, we use calculus rule by differentiating it with respect to ${k}_{1}$, ${k}_{2}$ and equating to zero, as following:

$$\frac{\partial MSE\left({T}_{P1}\right)}{\partial {k}_{1}}=2{\mu }_{y}^{2}{A}_{1}{k}_{1}-2{\mu }_{y}^{2}{C}_{1}-2{\mu }_{y} {E}_{1}{k}_{2}=0.$$

$$\frac{\partial MSE\left({T}_{P1}\right)}{\partial {k}_{2}}=-2{\mu }_{y}{E}_{1}{k}_{1}+2{B}_{1}{k}_{2}-2{\mu }_{y}{D}_{1}=0.$$

We get as system of equations given below:

22 $$\left[ {\begin{array}{*{20}c} {\mu_{y}^{2} A_{1} } & { - \mu_{y} E_{1} } \\ { - \mu_{y} E_{1} } & {B_{1} } \\ \end{array} } \right]\left[ {\begin{array}{*{20}c} {k_{1 } } \\ {k_{2} } \\ \end{array} } \right] = \left[ {\begin{array}{*{20}c} {\mu_{y}^{2} C_{1} } \\ {\mu_{y} D_{1} } \\ \end{array} } \right]$$

Using Cramer’s rule to solve the system of equations, we get:

$${k}_{1(opt)}=\frac{{B}_{1}{C}_{1}-{D}_{1}{E}_{1}}{{\Delta }_{1}},{k}_{2(opt)}=\frac{{\mu }_{y}\left({A}_{1}{D}_{1}+{C}_{1}{E}_{1}\right)}{{\Delta }_{1}}.$$

On the same line, we proceed for MSE of T_P2_ as following:

$$\frac{\partial MSE\left({T}_{P2}\right)}{\partial {k}_{3}}=2{\mu }_{y}^{2}{A}_{2}{k}_{3}-2{\mu }_{y}^{2}{C}_{2}-2{\mu }_{y} {E}_{2}{k}_{4}=0.$$

$$\frac{\partial MSE\left({T}_{P2}\right)}{\partial {k}_{4}}=-2{\mu }_{y}{E}_{2}{k}_{3}+2{B}_{2}{k}_{4}-2{\mu }_{y}{D}_{2}=0.$$

23 $$\left[ {\begin{array}{*{20}c} {\mu_{y}^{2} A_{2} } & { - \mu_{y} E_{2} } \\ { - \mu_{y} E_{2} } & {B_{2} } \\ \end{array} } \right]\left[ {\begin{array}{*{20}c} {k_{3 } } \\ {k_{4} } \\ \end{array} } \right] = \left[ {\begin{array}{*{20}c} {\mu_{y}^{2} C_{2} } \\ {\mu_{y} D_{2} } \\ \end{array} } \right]$$

We obtain:

$${k}_{3(opt)}=\frac{{B}_{2}\hspace{0.17em}{C}_{2}\hspace{0.25em} -\hspace{0.25em} {C}_{2}\hspace{0.17em}{E}_{2}}{{\Delta }_{2}}\hspace{0.25em} , {k}_{4(opt)}=\hspace{0.25em}\frac{ {C}_{2}\hspace{0.17em}{\mu }_{y}\hspace{0.17em}\left({A}_{2}+{E}_{2}\right)}{{\Delta }_{2}}$$

where, $${\Delta }_{1}={A}_{1}\hspace{0.17em}{B}_{1}\hspace{0.25em} -\hspace{0.25em} {{E}_{1}}^{2}, {\Delta }_{2}={A}_{2}\hspace{0.17em}{B}_{2}\hspace{0.25em} -\hspace{0.25em} {{E}_{2}}^{2}$$

Substituting the values of ${k}_{1}$​, ${k}_{2},$​ ${k}_{3}$ and ${k}_{4}$ into Eq. (20) and Eq. (21) respectively, yields the following expressions of minimum MSEs:

24 $$\mathop {{\text{min}}}\limits_{{k_{1} ,k_{2} }} MSE\left( {T_{P1} } \right) \approx {\upmu }_{y}^{2} \left( {1 - \frac{{A_{1} D_{1}^{2} + B_{1} C_{1}^{2} - 2C_{1} D_{1} E_{1} }}{{\Delta_{1} }}} \right)$$

25 $$\mathop {\min }\limits_{{k_{3} ,k_{4} }} MSE\left( {T_{P2} } \right) \approx {\upmu }_{y}^{2} \left( {1 - \frac{{C_{2}^{2} \left( {A_{2} \, + B_{2} \, - 2\,\,E_{2} } \right)}}{{\Delta_{2} }}} \right)$$

The proposed estimators are highly significant than their counterparts, which will be assessed numerically in the coming sections with different real as well as synthetic data sets.

## Efficiency comparison

The following criteria must be met for our suggested estimate to be more effective than the competing estimators: its mean square error (MSE) must be lower than that of the competing estimators.

### Conditions

From Eq. (1) and Eq. (24) and (25), The classical mean estimator ${\mu }_{y}$ will be outperformed by the suggested estimators if:

$$MSE\left({T}_{P\text{1,2}}\right)< MSE\left({T}_{0}\right)$$

or

$$\left[\left(\uplambda {C}_{y}^{2}-1\right)+{\theta }_{i}\right]>0$$

where ${\theta }_{i}=\frac{{A}_{i}{{D}_{i}}^{2}+{B}_{i}{{C}_{i}}^{2}-2{C}_{i}{D}_{i}{E}_{i}}{{A}_{i}{B}_{i}-{{E}_{i}}^{2}}$ , i = 1,2 corresponding to the first and second proposed estimator.

By comparing Eq. (2) and Eq. (24) and (25), $MSE\left({T}_{P\text{1,2}}\right)< MSE\left({T}_{1}\right)$ if:

$$\left[\left\{\uplambda \left({S}_{y}^{2}+\text{D}{S}_{x}^{2}-2\text{D}{S}_{xy}\right)-1\right\}+{\theta }_{i}\right]>0$$

By comparing Eq. (3) and Eq. (24), and (25) $MSE\left({T}_{P\text{1,2}}\right)< MSE\left({T}_{2}\right)$ if:

$$\left[\left\{\uplambda \left({S}_{y}^{2}+\text{D}{S}_{x}^{2}+2\text{D}{S}_{xy}\right)-1\right\}+{\theta }_{i}\right]>0$$

By comparing Eq. (4) and Eq. (24), and (25), $MSE\left({T}_{P\text{1,2}}\right)< MSE\left({T}_{3}\right)$ if:

$$\left[\left\{\uplambda \left({S}_{y}^{2}+\frac{1}{4}{D}^{2}{S}_{x}^{2}-D{S}_{xy}\right)-1\right\}+{\theta }_{i}\right]>0$$

By comparing Eq. (5), (7) and Eq. (24), and (25), $MSE\left({T}_{P\text{1,2}}\right)< MSE\left({t}_{\text{4,6}}\right)$ if:

$$\left[\left\{\uplambda {C}_{y}^{2}\left(1-{\rho }_{xy}^{2}\right)-1\right\}+{\theta }_{i}\right]>0$$

By comparing Eq. (6) and Eq. (24), and (25), $MSE\left({T}_{P\text{1,2}}\right)< MSE\left({T}_{5}\right)$ if:

$$\left[\left\{\uplambda \left({S}_{y}^{2}+{k}^{2}{D}^{2}{S}_{x}^{2}-2kD{S}_{yx}\right)-1\right\}+{\theta }_{i}\right]>0$$

By comparing Eq. (8) and Eq. (24), and (25), $MSE\left({T}_{P\text{1,2}}\right)< MSE\left({T}_{7}\right)$ if:

$$\left[\left\{C+\left(\frac{B{C}^{2}+A{D}^{2}-2CDE}{{E}^{2}-AB}\right)-1\right\}+{\theta }_{i}\right]>0$$

By comparing Eq. (9) and Eq. (24), and (25), $MSE\left({T}_{P\text{1,2}}\right)< MSE\left({T}_{8}\right)$ if:

$$\left[\left\{A+\left(\frac{B{E}^{2}+C{D}^{2}+2DEF}{{F}^{2}-BC}\right)-1\right\}+{\theta }_{i}\right]>0$$

The subsequent section confirm that these conditions hold true for all types of real data, when the main study variable is positively correlated with the auxiliary variable.

## Numerical illustration

Using PREs as the performance metric, empirical and simulated experiments are conducted to assess the effectiveness of the suggested and competing estimators. To assess the performance of the proposed and competing estimators, we have used PRE (Percent Relative Efficiency) as a performance index, given by

26 $$PRE\left( {T_{..} } \right) = \left( {\frac{{Var\left( {T_{0} } \right)}}{{MSE\left( {T_{..} } \right)}}} \right) \times 100$$

where *T*_*0*_ is the classical/usual estimator and ${T}_{..}$ are the competing and proposed estimators.

### Empirical study

**Fig. 1:**
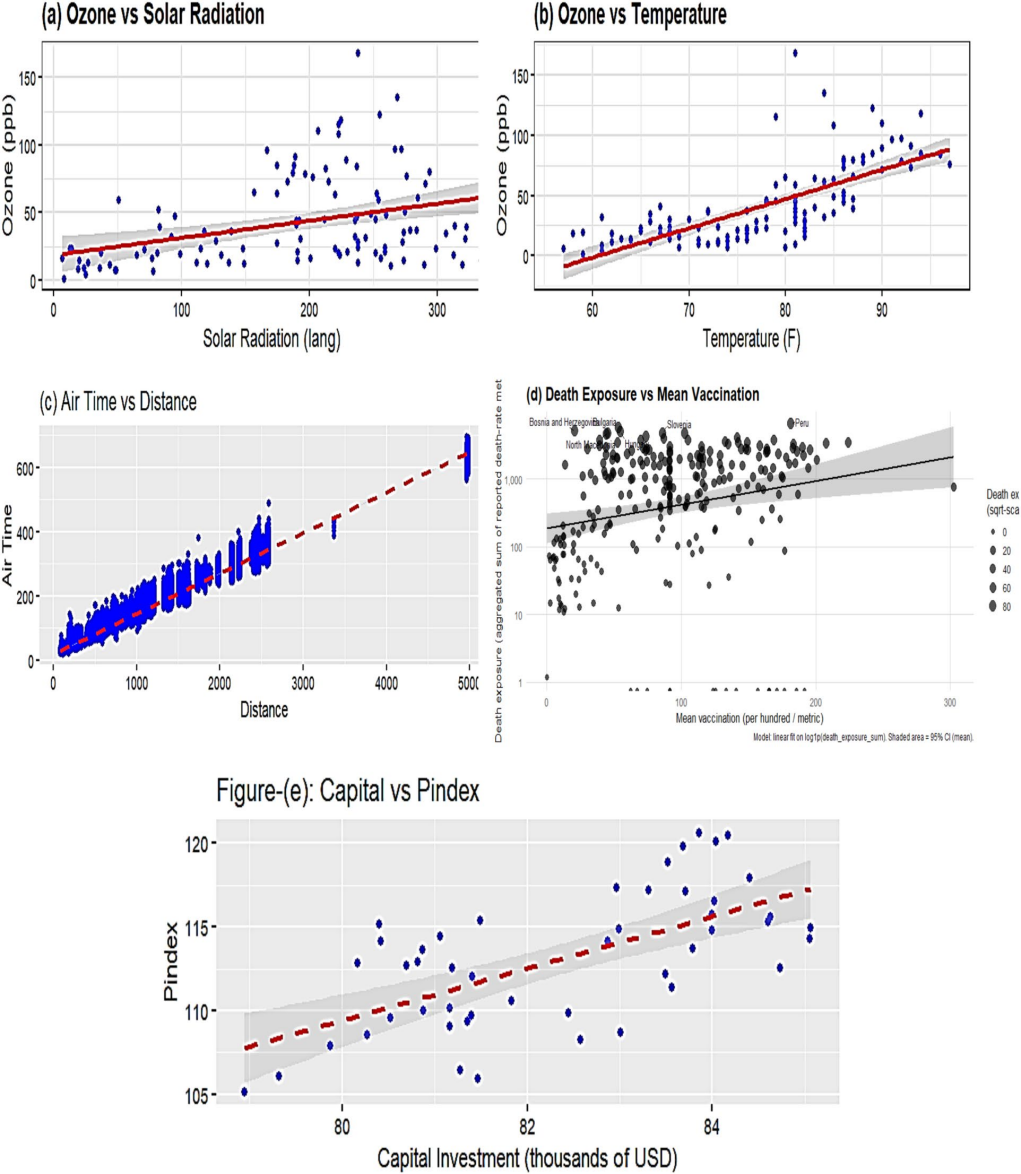
(**a–e**) Visualizing relationship between survey variable (Y) and auxiliary variable (X) across different data sets.

To assess the practical performance of the proposed logarithmic ratio–type estimators we conducted an empirical evaluation on five real-world engineering datasets. These datasets were chosen to represent a range of sample sizes, correlation structures, coefficients of variation, and degrees of skewness that commonly occur in engineering and environmental applications (see Table 1 for summary statistics). The datasets comprise: (i) Ozone vs. Solar Radiation [Source: Chambers^23^], (ii) Ozone vs. Temperature[Source: Chambers^23^], (iii) Air Time vs. Distance[Source: Wickham^24^], (iv) Total Deaths vs. Total Vaccinations[Source: Subramanian and Kumar^25^], and (v) Sales: Capital vs. Pindex[Source: Kadilar and Cingi^26^]. Each data source and its salient descriptive statistics (population/sample sizes, sample means, coefficients of variation, and sample correlation ${\rho }_{yx}$) are summarized in Table 1. Figure 1a–e shows scatterplots of the study variable.

**Table 1:** Summary Statistics of different datasets.

Source	Data-1	Data-II	Data-III	Data-IV	Data-V
N	327,346	4071	255	50	111
n	12	12	12	10	12
$\overline{X }$	1048	46,535	41,559.36	50,000	184.8018
$\overline{Y }$	150.7	1012.587	1227.28	2535.321	42.0991
$\rho$	0.99	0.7123	0.3003735	0.9825	0.3483
${C}_{y}$	0.621743	1.84	1.06	0.5887	0.79041
${C}_{x}$	0.7019	1.54	1.46	0.5830	0.49324
${C}_{yx}$	0.4320	2.01185	0.4665513	4276.0609	0.1357

**Fig. 2:**
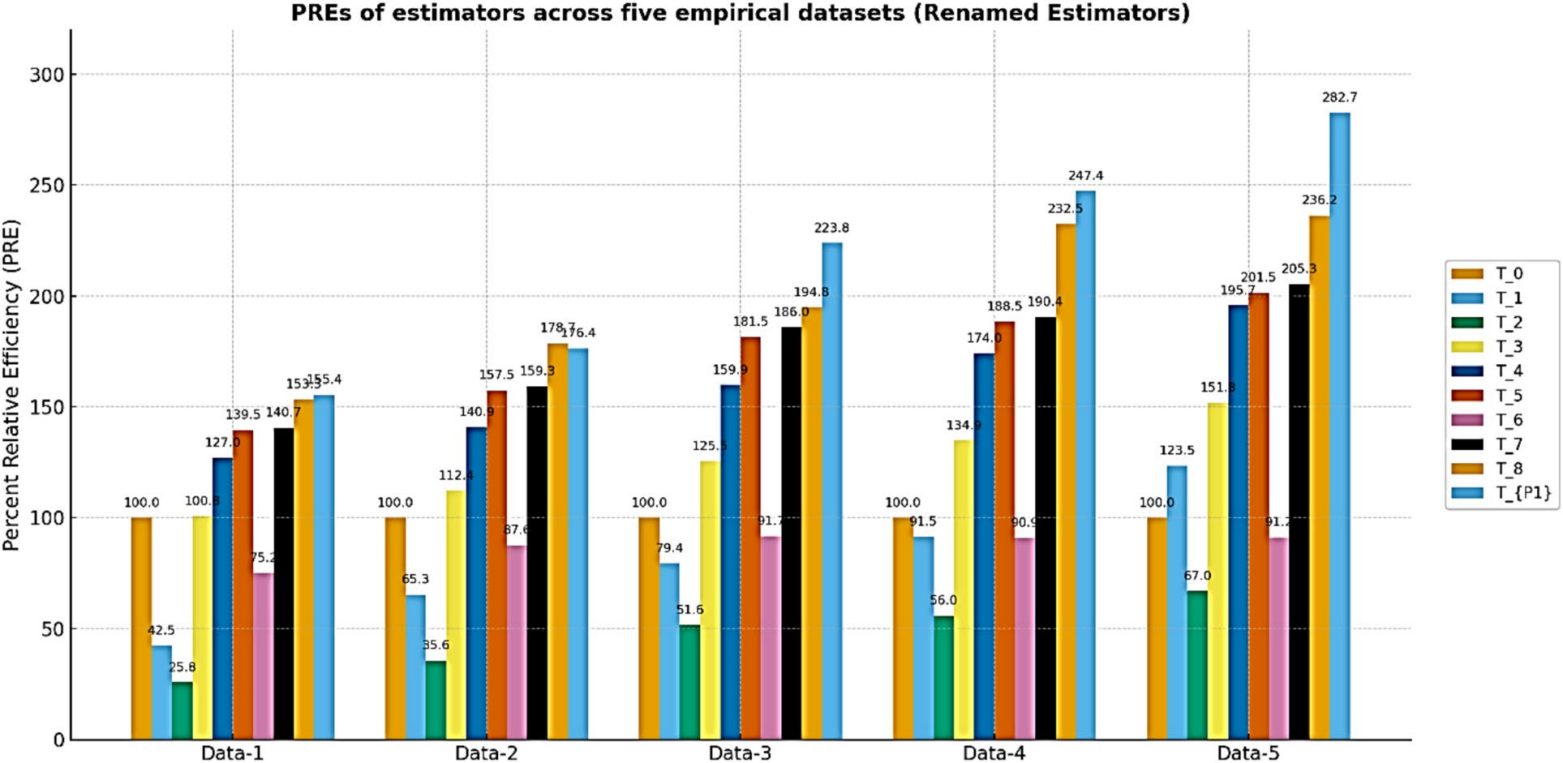
PREs of the proposed and competing estimators against the classical baseline estimator.

$Y$ against the auxiliary variable $X$ for the five empirical datasets (Fig. 1a: Ozone vs Solar Radiation; Fig. 1b: Ozone vs Temperature; Fig. 1c: Air Time vs Distance; Fig. 1d: Total Deaths vs Total Vaccinations; Fig. 1e: Sales: Capital vs Pindex), illustrating the varying strengths of association and distributional features that motivate the proposed log-ratio estimators. All empirical comparisons use simple random sampling without replacement (SRSWOR) as the design framework, and we evaluate estimator accuracy with Percent Relative Efficiency (PRE) relative to the classical sample mean:

Table 2 and its corresponding plot Fig. 2 show that the proposed estimators ${\text{T}}_{\text{P}1}$​ and ${\text{T}}_{\text{P}2}$​ consistently achieve the highest PRE-values across all five real datasets, with T_P2_​ reaching a peak of 282.7 on Data-5. The results confirm that the proposed estimators deliver superior efficiency in real-data applications compared to both the usual estimator and other traditional methods.

**Table 2:** PREs of the proposed and existing estimators relative to the baseline classical estimators across five real datasets.

Estimators	Data-1	Data-2	Data-3	Data-4	Data-5
*T*_*0*_	100	100	100	100	100
${T}_{1}$	42.478	65.341	79.436	91.486	123.505
${T}_{2}$	25.817	35.643	51.643	55.974	67.015
${\text{T}}_{3}$	100.816	112.359	125.489	134.856	151.774
${\text{T}}_{\text{4,7}}$	126.977	140.857	159.874	174.047	195.747
${\text{T}}_{5}$	139.54	157.472	181.490	188.484	201.539
${\text{T}}_{6}$	75.157	87.603	91.732	90.904	91.223
${\text{T}}_{8}$	140.67	159.292	186.032	190.447	205.342
T_P1_	153.33	178.745	194.848	232.484	236.226
T_P2_	155.36	176.356	223.847	247.384	282.678

### Simulation study

The simulation study was designed to (i) evaluate the finite-sample behaviour and stability of the proposed logarithmic ratio–type estimators (*T*_*P1*_, *T*_*P2*_ under controlled distributional regimes, (ii) compare their performance with a broad set of existing estimators (classical mean, ratio/product, exponential and other transformed forms), and (iii) verify whether the first-order analytical bias/MSE formulas and the closed-form optimal constants derived in Sect. 3 provide realistic guidance for finite samples. Synthetic finite populations size N = 1000 were generated from three distributional families that together span the canonical sampling regimes practitioners face: Multivariate Normal (symmetric, light tails), Gamma (positively skewed), and Lognormal (heavy-tailed). These choices allow assessment of estimator robustness to skewness and heavy tails. For each family we constructed three bivariate populations differing only in the study–auxiliary correlation ${\rho }_{yx}\in (0.92,,0.71,,0.38)$ (labelled Data-1, Data-2 and Data-3 respectively). All populations share a common mean vector and a covariance structure given by:

$$\mu =\left[\begin{array}{c}40\\ 50\end{array}\right],\Sigma =\left[\begin{array}{cc}1& {\rho }_{yx}\\ {\rho }_{yx}& 1\end{array}\right]$$

The core experiments draw repeated SRSWOR samples of sizes n ε50, 150, 200 from each synthetic finite population; additional sensitivity runs that include smaller $n$ (e.g. $n=20$) and intermediate values were also performed to check small-sample behavior. Each simulation was repeated 10,000 times in RStudio, and estimator performance was evaluated using Percent Relative Efficiency (PRE), defined as:

$${\text{PRE}}\left( {T_{i} } \right) = \frac{{{\text{Var}}\left( {T_{0} } \right)}}{{{\text{MSE}}\left( {T_{i} } \right)}} \times 100$$

Where *T*_*0*_​ denotes the reference estimator and ${T}_{i}$ represents the ${i}^{th}$ competing estimator. The variance and mean squared error (MSE) were computed over the 10,000 replications as:

27 $${\text{Var}}\left( {T_{0} } \right) = \frac{{\mathop \sum \nolimits_{r = 1}^{R} \left( {T_{0}^{\left( r \right)} - \mu_{y} } \right)^{2} }}{R},\quad {\text{MSE}}\left( {T_{i} } \right) = \frac{{\mathop \sum \nolimits_{r = 1}^{R} \left( {T_{i}^{\left( r \right)} - \mu_{y} } \right)^{2} }}{R}$$

where *R* = 10,000 is the number of simulation replications.

The mean PRE-values across all replications and sample sizes were recorded and summarized in Table 3 to evaluate estimator efficiency under different distributional and correlation scenarios.

**Table 3:**
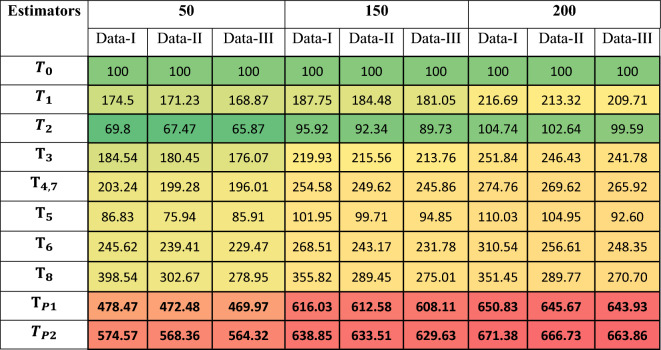
Heatmap table of PREs of the proposed and competing Estimators against the classical estimator for Simulated Data-1 when** ρ**_yx_ is high Correlation.

**Fig. 3:**
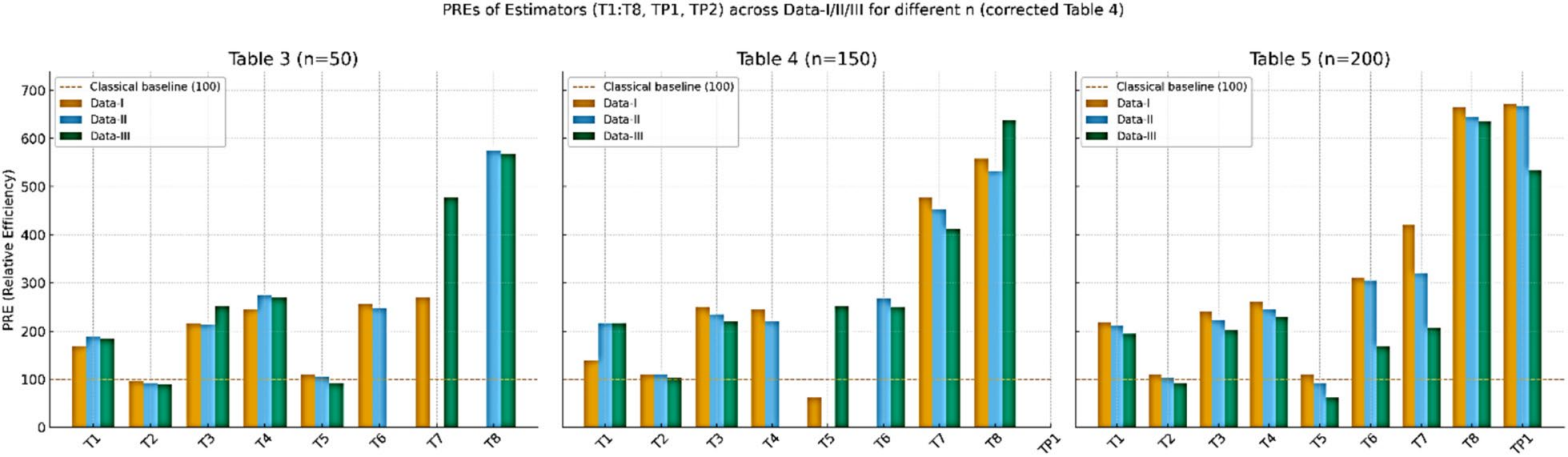
visualization of PREs of the proposed and competing estimators against the classical estimators for different setup of sample size and correlation levels for Data-I(MV-Normal), Data-II(Gamma), and Data-III(Lognormal).

Table To further evaluate the robustness of the proposed estimators under non-normal and positively skewed settings, we conducted a simulation study using a Gamma-distributed finite population with a strong positive auxiliary correlation ρ ≈ 0.90. Figure 3 presents bar-plots of the replicate-wise percent relative efficiency (PRE) for all considered estimators relative to the classical estimator *T*_*0*_​ across three sample sizes n ε 50, 150, 200. For each scenario, finite-population characteristics were employed to compute the tuning constants, and optimal parameters for ​ *T*_*7*_and *T*_*8*_​ were obtained via a data-driven numerical minimization of the empirical mean-squared error. The results demonstrate a clear and consistent dominance of the proposed estimators T_P1_​ and T_P2_, both in terms of median PRE and distributional stability, especially as the sample size increases. The remaining logarithmic and exponential-type estimators also exhibit improved performance over the classical mean estimator; however, they are comparatively less stable and efficient than the proposed class. These findings reinforce the efficacy of the proposed estimators under skewed populations and high-correlation structures.

In Tables 6,7,8, we report uncertainty for PREs via both Monte-Carlo standard errors and nonparametric percentile confidence intervals (CI). For simulations we compute per-replicate PREs across RRR Monte-Carlo replicates and report the mean PRE with its Monte-Carlo standard error $(SE = sd(PRE)/\sqrt R )$together with the empirical 95% percentile CI (2.5%–97.5% quantiles). For empirical/finite-population analyses, repeated SRSWOR resampling from the known population (or a design-consistent bootstrap when the population is not fully available) was used to estimate MSEs and PREs; the resulting replicate PREs are summarized with mean (± MC SE) and percentile CI.

Extremely high PRE-values and wide CIs for classical ratio/product-type estimators *T*_*1*_–*T*_*8*_ reflect instability under skewed Gamma populations, especially with small samples, as revealed by Table 3, 4 and 5. In contrast, the proposed log-ratio estimators *T*_*P1*_, *T*_*P2*_​ demonstrate stable PRE, narrow confidence intervals, and robust behavior, confirming their suitability for non-normal data structures (Table 8). The proposed estimators therefore offer a computationally efficient and versatile framework for modern sampling applications, particularly where skewness, auxiliary information, and finite-population structures play a central role.

**Table 4:**
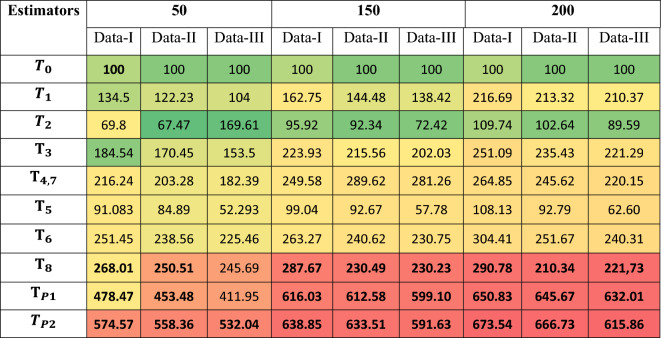
Heatmap table of PREs of the proposed and alternative estimators relative to the classical estimator for Simulated Data-2 under moderate *ρ*_yx_.

**Table 5:**
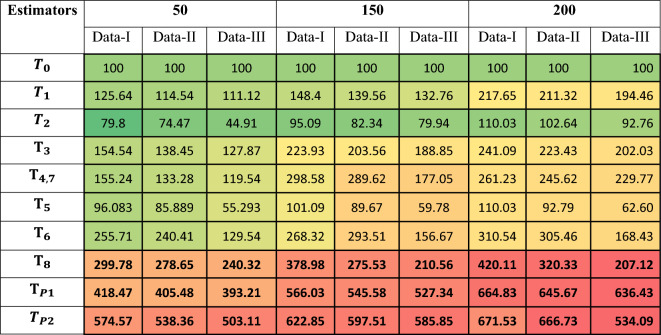
Heatmap table of PREs of the proposed and alternative estimators relative to the classical estimator for Simulated Data-3 under low correlation *ρ*_yx_.

## Results and discussion

**Fig. 4:**
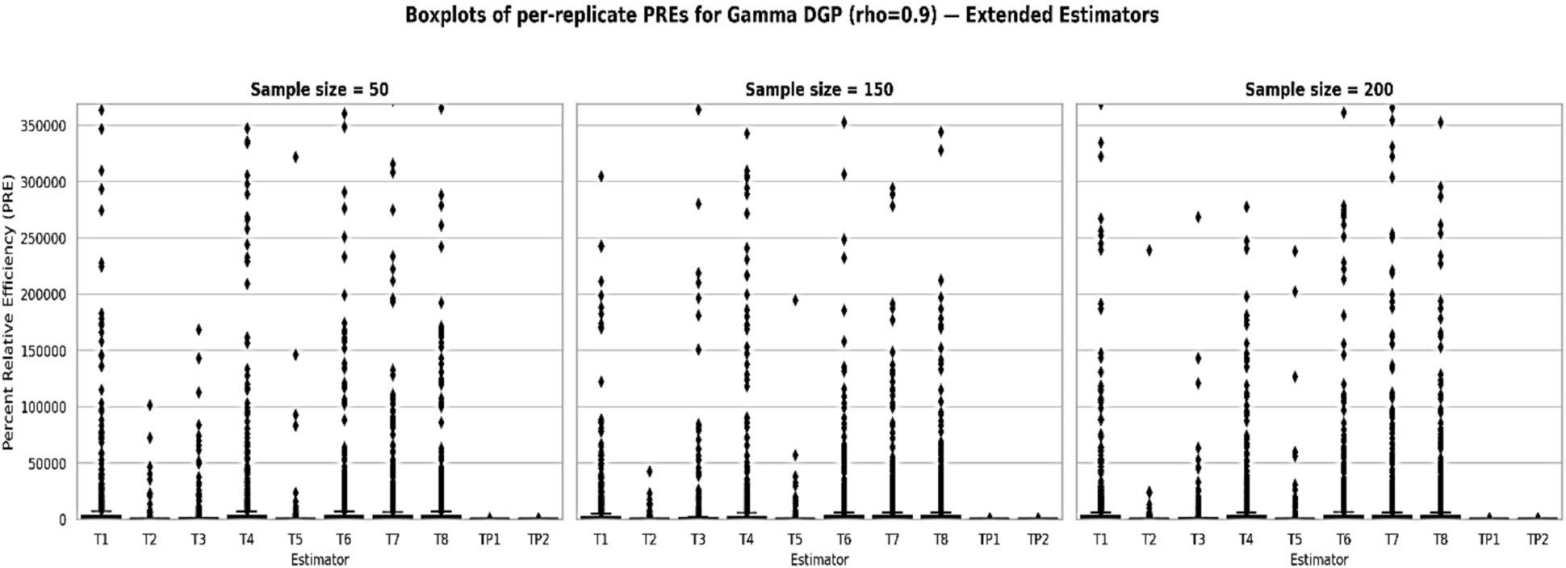
Box-plots of percent relative efficiency (PRE) for proposed and competing estimators under a Gamma population with strong auxiliary correlation ρ ≈ 0.90 and sample sizes n = 50,150,200. Dots represent mean PRE; vertical lines denote 95% Monte-Carlo confidence intervals.

In this article, we developed two log-ratio type estimators of finite population mean under SRSWOR scheme. We derived first order Bias and MSE for both estimators to theoretically validate our methodology. The empirical and simulation evidence together provide a consistent and interpretable picture of the proposed estimators’ performance. Table 1 contextualizes the real datasets by summarizing sample sizes, variability and correlation structures, and Table 2 shows that the proposed log-ratio estimators achieve uniformly higher percent-relative-efficiency (PRE) than the classical mean across all five empirical datasets. The simulation tables (Tables 3–8) systematically confirm these gains: as sample size increases from n = 50 to n = 200 the PRE of the proposed estimators rises markedly and they maintain top ranking across Data-I/II/III, with the corrected values for Table 4 removing parsing artefacts and reinforcing the same conclusion. Monte-Carlo summaries (Tables 6, 7, 8) quantify the uncertainty around mean PREs and show that estimator spread and Monte-Carlo standard errors shrink with larger n, indicating both higher efficiency and greater stability. Figures 1a–e visualizes the study–auxiliary relationships that motivate auxiliary-based adjustments, Fig. 2 highlights the empirical PRE advantages, and the box-plots in Fig. 4 demonstrate that the proposed estimators not only improve median PRE but also yield substantially narrower replicate-wise distributions under skewed Gamma populations. Finally, the three-panel grouped figure (n = 50, 150, 200) provides a direct visual comparison across sample sizes and confirms that the proposed estimators deliver robust, monotonic improvements in efficiency as sample size and correlation increase, making them practically attractive for survey and engineering applications where auxiliary information is available.The proposed estimators reduce to or improve upon several established estimators under special parameter choices, confirming theoretical coherence. Relative ranking is stable across scenarios: the two proposed forms dominate classical, product, and exponential competitors in both empirical and simulated environments.

**Table 6:** Percent relative efficiency (pre) of competing and proposed estimators under gamma population with strong auxiliary correlation ρ ≈ 0.90, and sample size n=150.

Estimator	Mean	Normal		Percentile	
		Lowe confidence limit(LCL)	Upper confidence limit (UCL)	Lowe confidence limit (LCL)	Upper confidence limit (UCL)
*T*_*0*_	100.00	100.00	100.00	100.00	100.00
*T*_*1*_	3.970e + 04	2.250e + 03	7.710e + 04	1.320e + 00	1.430e + 05
*T*_*2*_	2.480e + 03	– 1.840e + 03	6.810e + 03	1.530e + 00	9.970e + 02
*T*_*3*_	5.670e + 03	– 1.030e + 02	1.140e + 04	5.070e + 00	1.430e + 04
*T*_*4*_	1.810e + 07	– 1.650e + 07	5.260e + 07	1.940e + 00	9.060e + 05
*T*_*5*_	2.690e + 04	– 1.690e + 04	7.070e + 04	5.460e + 00	1.230e + 04
*T*_*6*_	1.090e + 08	– 1.040e + 08	3.210e + 08	1.580e + 00	4.390e + 05
*T*_*7*_	4.110e + 04	– 3.770e + 03	8.610e + 04	2.260e + 00	7.970e + 04
*T*_*8*_	4.360e + 04	– 8.170e + 03	9.530e + 04	2.290e + 00	9.540e + 04
*T*_*P1*_	3.180e-06	2.700e-06	3.660e-06	3.170e-09	1.400e-05
*T*_*P2*_	3.720e-03	3.150e-03	4.290e-03	3.690e-06	1.680e-02

**Table 7:** Percent Relative Efficiency (PRE) of competing and proposed estimators under Gamma population with strong auxiliary correlation ρ ≈ 0.90, and sample size n = 150.

Estimator	Mean	Normal		Percentile	
		LCL	UCL	LCL	UCL
*T*_*0*_	1.000e + 02	1.000e + 02	1.000e + 02	1.000e + 02	1.000e + 02
*T*_*1*_	7.150e + 05	– 9.150e + 04	1.520e + 06	3.100e-01	4.290e + 05
*T*_*2*_	5.140e + 03	– 9.170e + 02	1.120e + 04	4.100e-01	2.000e + 03
*T*_*3*_	1.070e + 04	– 1.160e + 03	2.240e + 04	5.300e-01	4.390e + 03
*T*_*4*_	1.090e + 05	– 1.010e + 04	2.290e + 05	2.600e-01	1.870e + 05
*T*_*5*_	8.870e + 03	– 1.630e + 03	1.940e + 04	5.300e-01	1.020e + 04
*T*_*6*_	1.430e + 05	– 1.850e + 04	3.050e + 05	5.000e-01	2.630e + 05
*T*_*7*_	6.470e + 04	– 6.180e + 03	1.350e + 05	3.900e-01	5.610e + 04
*T*_*8*_	1.180e + 04	2.160e + 03	2.150e + 04	4.100e-01	4.260e + 04
*T*_*P1*_	1.200e-07	9.970e-08	1.390e-07	3.930e-11	4.920e-07
*T*_*P2*_	1.260e-03	1.050e-03	1.470e-03	4.170e-07	5.230e-03

**Table 8:** Percent relative efficiency (PRE) of competing and proposed estimators under Gamma population with strong auxiliary correlation ρ ≈ 0.90, and sample size n = 200.

Estimator	Mean	Normal		Percentile	
		LCL	UCL	LCL	UCL
*T*_*0*_	1.000e + 02	1.000e + 02	1.000e + 02	1.000e + 02	1.000e + 02
*T*_*1*_	3.810e + 05	– 3.070e + 05	1.070e + 06	1.060e + 00	1.030e + 05
*T*_*2*_	1.140e + 03	2.150e + 02	2.060e + 03	8.700e-01	5.390e + 03
*T*_*3*_	1.200e + 04	– 3.170e + 03	2.710e + 04	3.720e + 00	8.850e + 03
*T*_*4*_	3.890e + 04	4.010e + 03	7.380e + 04	1.290e + 00	1.720e + 05
*T*_*5*_	3.560e + 03	– 6.270e + 02	7.740e + 03	3.960e + 00	1.630e + 04
*T*_*6*_	7.980e + 04	– 2.580e + 04	1.850e + 05	1.320e + 00	7.870e + 04
*T*_*7*_	1.960e + 06	– 1.740e + 06	5.660e + 06	1.070e + 00	3.090e + 05
*T*_*8*_	1.380e + 05	– 3.840e + 04	3.150e + 05	1.050e + 00	3.270e + 05
*T*_*P1*_	4.840e-08	4.080e-08	5.600e-08	3.450e-11	2.590e-07
*T*_*P2*_	9.140e-04	7.700e-04	1.060e-03	6.440e-07	4.890e-03

## Conclusion

We propose two logarithmic ratio–type estimators, T_P1_ and T_P2_, for finite-population mean estimation under simple random sampling without replacement (SRSWOR). Closed-form first-order expressions for bias and mean squared error (MSE) are derived and used to obtain analytic tuning constants. Empirical evaluation on five engineering datasets and extensive Monte-Carlo experiments (multiple distributions, correlations, and sample sizes) shows that the proposed estimators yield consistent and often large percent-relative-efficiency (PRE) gains over the classical sample mean and common competitors; gains increase with sample size and with positive study–auxiliary correlation. The estimators are computationally simple to implement and, when the auxiliary mean is known and the auxiliary variable is positively correlated with the study variable, offer a practical route to substantially reduced MSE and smaller required sample sizes for a given precision.

We also identify important limitations: the current theory assumes SRSWOR, known auxiliary means, and positive correlation between study and auxiliary variables, conditions that facilitate closed-form analysis but may be restrictive in some applied settings. To widen applicability, future research should (i) relax the known-mean assumption via calibration, model-assisted, or empirical-Bayes corrections; (ii) adapt the estimators to stratified and two-phase sampling by incorporating stratum- or phase-specific adjustment factors; (iii) derive higher-order bias/MSE approximations and robust variance estimators for small-sample inference; and (iv) address practical data issues such as nonresponse and measurement error. Finally, while our focus is parameter estimation rather than prediction, the efficiency principles developed here could be embedded within parameter-estimation components of machine-learning pipelines (e.g., regression weighting, EM steps) to improve stability and predictive performance. Overall, T_P1_ and T_P2_ provide a theoretically grounded and practically useful improvement for mean estimation when reliable auxiliary information is available.

## Data Availability

The data used and analyzed in this article are available in the published article as cited against each data sets in the numerical section of this article.

## List of symbols

N: Size of the population
n: Size of the sample
$$S_{y}^{2}$$: Variance of y
$$S_{x}^{2}$$: Variance of x
$$s_yx$$: Covariance
$C_y$: Coefficient of variation (y)
$C_x$: Coefficient of variation (x)
$C_yx$: Coefficient of covariance x, y
$$\alpha \,and\,\beta$$: Generalizing constants
$e_0,e_1$: Relative error in $\bar{y} = \frac{{\bar{y} - \mu _{y} }}{{\mu _{y} }}$ and $\bar{x} = \frac{{\bar{x} - \mu _{x} }}{{\mu _{x} }}$respectively
$S_{y}^{2}$: Population variance of (y)
$S_{x}^{2}$: Population variance of (x)
$\rho _{{yx}}$: Population correlation coefficient
$\bar{Y}$: Mean of the population of y
$\bar{X}$: Population Mean of x
R: Population ratios
f: Sampling fraction
$T_{{ln_{1} }}$: First proposed estimator
$T_{{ln_{2} }}$: Second proposed estimator
$$k_{1} ,~k_{2} ,~k_{3} ,~k_{4}$$: Optimizing constants
